# Small Heat Shock Proteins Are Novel Common Determinants of Alcohol and Nicotine Sensitivity in *Caenorhabditis elegans*

**DOI:** 10.1534/genetics.115.185025

**Published:** 2016-01-13

**Authors:** James R. Johnson, Dayani Rajamanoharan, Hannah V. McCue, Kim Rankin, Jeff W. Barclay

**Affiliations:** The Physiological Laboratory, Institute of Translational Medicine, University of Liverpool, Liverpool L69 3BX, United Kingdom

**Keywords:** HSF1, alpha crystallin, heat shock proteins, alcohol, nicotine

## Abstract

Addiction to drugs is strongly determined by multiple genetic factors. Alcohol and nicotine produce distinct pharmacological effects within the nervous system through discrete molecular targets; yet, data from family and twin analyses support the existence of common genetic factors for addiction in general. The mechanisms underlying addiction, however, are poorly described and common genetic factors for alcohol and nicotine remain unidentified. We investigated the role that the heat shock transcription factor, HSF-1, and its downstream effectors played as common genetic modulators of sensitivity to addictive substances. Using *Caenorhabditis elegans*, an exemplary model organism with substance dose-dependent responses similar to mammals, we demonstrate that HSF-1 altered sensitivity to both alcohol and nicotine. Using a combination of a targeted RNAi screen of downstream factors and transgenic approaches we identified that these effects were contingent upon the constitutive neuronal expression of HSP-16.48, a small heat shock protein (HSP) homolog of human α-crystallin. Furthermore we demonstrated that the function of HSP-16.48 in drug sensitivity surprisingly was independent of chaperone activity during the heat shock stress response. Instead we identified a distinct domain within the N-terminal region of the HSP-16.48 protein that specified its function in comparison to related small HSPs. Our findings establish and characterize a novel genetic determinant underlying sensitivity to diverse addictive substances.

THE most commonly used pharmacological addictive substances are alcohol and nicotine and the economic burden from abuse of these substances is extremely high. Indeed, tobacco and alcohol use account for 6 million and 2.5 million deaths per year, respectively (World Health Organization 2010, 2011). Although environmental factors play a role in addiction, heritability estimates for nicotine and alcohol range between 30 and 60% (Bierut 2011; Agrawal *et al.* 2012; Wang *et al.* 2012; Demers *et al.* 2014; Buhler *et al.* 2015). One factor thought to play a role in the acquisition of substance dependence is an individual’s initial level of response to the drug, which is itself genetically influenced (Schuckit *et al.* 2004; Schuckit *et al.* 2011, 2012). Nicotine acts physiologically as a nervous system stimulant through direct binding and activation of nicotinic acetylcholine receptors (Dani and Balfour 2011). Additional factors that influence nicotine sensitivity have been identified, such as transient receptor potential (TRP) channels (Feng *et al.* 2006; Talavera *et al.* 2009). In contrast to nicotine, alcohol is a nervous system depressant thought to function by low-affinity interactions with specific target proteins (Howard *et al.* 2011; Trudell *et al.* 2014), such as protein kinase C (Newton and Ron 2007; Das *et al.* 2009), or membrane receptors and ion channels, for example GABA_A_ receptors (Aryal *et al.* 2009; Bodhinathan and Slesinger 2013; Howard *et al.* 2014). Although many modulators of alcohol sensitivity have been identified (Davies *et al.* 2003; Kapfhamer *et al.* 2008; Pietrzykowski *et al.* 2008; Barclay *et al.* 2010; Kaun *et al.* 2012), our understanding of acute alcohol action within the nervous system remains incomplete.

Genome-wide association studies (GWAS) on nicotine and alcohol dependence behaviors have identified potential contributing factors (Bierut 2011; Agrawal *et al.* 2012; Wang *et al.* 2012; Demers *et al.* 2014; Buhler *et al.* 2015) often reinforcing the link between modulators of substance efficacy or sensitivity and addictive predisposition. For alcohol, contributing factors reliably identified are enzymes involved in its metabolism, such as alcohol and aldehyde dehydrogenases (Edenberg *et al.* 2006; Frank *et al.* 2012; Gelernter *et al.* 2014; Quillen *et al.* 2014) as well as direct pharmacological targets such as GABA_A_ receptors (Bierut *et al.* 2010). For nicotine dependence, GWAS studies have also identified metabolic enzymes (Thorgeirsson *et al.* 2008) as well as the endogenous pharmacological target for nicotine, the nicotinic acetylcholine receptors (Bierut *et al.* 2007; Liu *et al.* 2010; Kapoor *et al.* 2012). Despite explicit pharmacological differences between addictive substances, such as alcohol and nicotine, data from family and twin analyses support the contribution of unidentified common genetic factors underlying substance dependence (Funk *et al.* 2006; Bierut 2011; Agrawal *et al.* 2012). Identification of these common factors, therefore, is a key unresolved question in addiction research and of considerable clinical and therapeutic importance.

Exposure of cells, tissues, or organisms to a stressful or harmful environment can activate the heat shock response, an upregulation in the expression of members of the heat shock protein (HSP) family of cellular chaperones. Indeed acute exposure to stressful concentrations of ethanol in *Caenorhabditis elegans* increases expression of a small number of genes, in particular a subset of HSPs (Kwon *et al.* 2004) and acute alcohol addition to cultured mouse neurons also induces HSP expression (Pignataro *et al.* 2007). Control over HSP expression, under both basal and stressful conditions, is governed by the heat shock transcription factor (HSF) (Anckar and Sistonen 2011). Here we characterize that HSF-1 is a codeterminant of both alcohol and nicotine sensitivity in *C. elegans* and that this phenotype requires the small HSP, HSP-16.48, a homolog of human α-crystallin. We show further that HSP-16.48 function in drug sensitivity is surprisingly unrelated to a chaperone action during the heat shock stress response. Finally we identify precisely the domain within its N-terminal region that determines the specificity of HSP-16.48 function compared to other closely related small HSPs. These results present a novel potential explanation for the common genetic basis underlying addiction.

## Materials and Methods

### Nematode culture, strains, and genetics

*C. elegans* strains were grown under standard conditions on nematode growth medium (NGM) agar plates at 20° with *Escherichia coli* OP50 as a food source as previously described (Graham *et al.* 2009; Johnson *et al.* 2009; Edwards *et al.* 2012). The following strains were used in this study: Bristol N2 (wild type), *hsf-1 (sy441)*, *hsp-3 (ok1083)*, *hsp-16.1/hsp-16.48 (ok577)*, and *hsp-16.11/hsp-16.49 (tm1221)*. To analyze potential effects of *pat-10* overexpression, we used the AGD1101 strain (Baird *et al.* 2014). To analyze potential alterations in muscle or neuronal morphology, we utilized, respectively, the DM8005 strain containing a GFP-tagged *myo-3* protein (Meissner *et al.* 2009) and the NM306 strain containing a GFP-tagged *snb-1* protein (Nonet 1999). Transgenic strains were generated by germline injection (Graham *et al.* 2009; Johnson *et al.* 2009; Edwards *et al.* 2012). For each transgenic strain, three individual independently derived transgenic lines were isolated and analyzed; the results presented here were consistent for all generated lines; however, individual line results can be found in Supporting Information, Table S2. The transgenic strains used in this study were: *hsf-1 (sy441);Ex[P_hsf-1_*::*hsf-1]*, *hsf-1 (sy441);Ex[P_rab-3_*::*hsf-1]*, *hsf-1 (sy441);Ex[P_myo-3_*::*hsf-1]*, *hsf-1 (sy441);Ex[P_vha-6_*::*hsf-1]*; *hsf-1 (sy441);Ex[P_rab-3_*::*hsp-16.48]*; N2;*Ex[P_hsf-1_*::*hsf-1]*; N2;*Ex[P_rab-3_*::*hsp-16.48]*, N2;*Ex[P_rab-3_*::*hsp-16.48ΔN]* (HSP-16.48_AA54-143_), N2;*Ex[P_rab-3_*::*hsp-16.48ΔC]* (HSP-16.48_AA1-128_), N2;*Ex[P_rab-3_*::*hsp-16.48ΔNC]* (HSP-16.48_AA54-128_), N2;*Ex[P_rab-3_*::*hsp-16.48Δ38-44]*, N2;*Ex[P_rab-3_*::*hsp-16.1 chimera]* (a fusion construct of the N terminus of *hsp-16.48* (HSP-16.48_AA1-70_) with the crystallin domain and C-terminus of *hsp-16.1* (HSP-16.1_AA67-145_)), N2;*Ex[P_rab-3_*::*hsp-16.48 E118N]*; N2;*Ex[P_hsf-1_*::*hsp-16.48]*, N2;*Ex[P_hsf-1_*::*hsp-16.1]*, and N2;*Ex[P_hsf-1_*::*hsp-1]*. For study of HSP-16.48 expression, we generated and examined green fluorescent protein (GFP) expression in *unc-18 (e81);[P_hsp-16.48_*::*GFP+P_unc-18_*::*unc-18]*.

### RNA interference screen

To perform the RNAi screen, we used the *rrf-3 (pk1426)* strain, as it has been widely characterized and has enhanced efficiency in neurons (Zhuang and Hunter 2011). In both cases, RNAi was performed by feeding (Kamath and Ahringer 2003; Kamath *et al.* 2003) using the ORFeome-based RNAi library (Rual *et al.* 2004). Briefly, HT115 RNAi bacteria were cultured in LB media containing 100 µg/ml ampicillin and then spotted in three 50-µl drops onto 60-mm diameter NGM plates containing 1 mM isopropyl β-1-thiogalactopyranoside (IPTG) and 25 µg/ml carbenicillin. NGM plates were dried at least 4 days before seeding. To each RNAi plate, five L3–L4 worms were added and cultured at 20°. Phenotypic analysis was performed on first generation progeny (days 1–2, adult hermaphrodites) fed with the indicated individual RNAi bacterial clones at 20°. For each feeding clone, the locomotion rate of animals was quantified as thrashing in Dent’s solution (ethanol experiments) or body bends on unseeded NGM agar plates (nicotine experiments) as defined below in behavioral assays. Negative controls for the RNAi screen were feeding with the empty feeding vector and RNAi of an HSP unrelated to the heat shock stress response, the endoplasmic reticulum chaperone *hsp-3*. Positive controls for the RNAi ethanol screen were feeding with clones targeting *rab-3* and *slo-1* both of which would be predicted from mutant analysis to decrease ethanol sensitivity (Davies *et al.* 2003; Kapfhamer *et al.* 2008). Positive controls for the RNAi nicotine screen were feeding with clones targeting *trp-2* and *acr-15* both of which would be predicted from mutant analysis to decrease nicotine sensitivity (Feng *et al.* 2006).

### Molecular cloning

With the exception of *P_rab-3_* (kind gift of M. Nonet, Washington University, St. Louis), which is described previously (Edwards *et al.* 2012), and *P_myo-3_* (pPD133.61) (kind gift of A. Fire, Stanford University, Stanford, CA), tissue and cell type-specific promoters were amplified from Bristol N2 genomic DNA and cloned into pPD117.01 (kind gift of A. Fire) in place of *P_mec-7_*. The following primers were used for promoter cloning:

*P_hsf-1_* forward: ACTGGCGCGCCGGAATGCTATTTCGCAATCACG
*P_hsf-1_* reverse: AGTCGGATCCTTTACGAACTAGCACGCGGTATC
*P_vha-6_* forward: GTAAGGCGCGCCAAGTTGAACCATGTATGGGAACCG
*P_vha-6_* reverse: AAAGGGATCCGGGTTTTGGTAGGTTTTAGTCG
*P_hsp-16.48_* forward: GTAAGGCGCGCCGATTGTAGTTTGAAGATTTCACAATTAGAGTG
*P_hsp-16.48_* reverse: GCATGGATCCTCTTGAAGTTTAGAGAATGAACAGTAAGC.

These constructs were converted to Gateway DEST vectors with the use of a conversion cassette (Life Technologies). Genes of interest were amplified from N2 genomic DNA and cloned into pDONR201 and recombined into these DEST vectors to produce tissue-specific expression vectors. The following primers were used for gene cloning:

*hsf-1* attB forward: GGGGACAAGTTTGTACAAAAAAGCAGGCTTCAAAATGCAGCCAACAGGGAATCAA
*hsf-1* attB reverse: GGGGACCACTTTGTACAAGAAAGCTGGGTCTTAAACCAAATTAGGATCCGATGG
*hsp-16.48* attB forward: GGGGACAAGTTTGTACAAAAAAGCAGGCTTCATGCTCATGCTCCGTTCTCC
*hsp-16.48* attB reverse: GGGGACCACTTTGTACAAGAAAGCTGGGTCAGATTAATGTTTTGCAACAAAATTAATGGG
*hsp-16.1* attB forward: GGGGACAAGTTTGTACAAAAAAGCAGGCTTCATGTCACTTTACCACTATTTCCGTC
*hsp-16.1* attB reverse: GGGGACCACTTTGTACAAGAAAGCTGGGTCTTATTCAGAAGTTTTTTGTTCAACGGG
*hsp-1* attB forward: GGGGACAAGTTTGTACAAAAAAGCAGGCTTCAAAATGAGTAAGCATAACGCTGTTG
*hsp-1* attB reverse: GGGGACCACTTTGTACAAGAAAGCTGGGTCAGATTAGTCGACCTCCTCGATCG.

### RNA extraction and quantitative real-time PCR

A total of 100 worms were picked into 100 μl of nuclease-free water, mixed with an additional 400 μl of TRIzol (Invitrogen), and frozen at −80°. Following one freeze–thaw cycle, worm samples were disrupted and homogenized on a vortex shaker at 4° for 40 min. A further 200 μl of TRIzol was added and vortexed before addition of 140 μl of chloroform. Samples were then centrifuged at 12,000 × *g* at 4° for 15 min. Following two separations of the aqueous upper phase with chloroform, RNA was precipitated by dropwise addition of equal volume 70% ethanol made in nuclease-free water. RNA was then treated with RNAse-free DNAse I (Qiagen) and then purified using the RNeasy Mini kit (Qiagen) according to the manufacturer’s instructions. RNA concentration and purity were quantified using a NanoDrop 1000 spectrophotometer (Thermo Fisher Scientific, Waltham). First-strand cDNA was synthesized using the ProtoScript First Strand cDNA Synthesis kit (New England Biolabs) using equal amounts of RNA and the random primer mix according to the manufacturer’s instructions. The cDNA was diluted (1:50) with diethylpyrocarbonate (DEPC)-treated water and stored at −80° until required. For gene expression analysis, master mixes for diluted cDNA samples mixed with DEPC water and selected qRT-PCR primers mixed with iTaq Universal SYBR Green Supermix (BioRad) were made and added to 96-well plates sequentially to make a final reaction volume of 10 μl per well. Reactions were performed using an IQ5 real-time PCR detection system (Bio-Rad). The thermocycler was programmed to first heat to 95° for 3 min, followed by 40 cycles of a 10-sec denaturation step at 95°, a 30-sec annealing step at 58°, and a 30-sec elongation step at 72°. qPCR reactions were carried out with three technical repeats of three individual biological replicates and transcript expression was analyzed using Bio-Rad CFX Manager 3.0 software using the comparative Ct method. Internal controls were the geometric mean of *cdc-43*, *pmp-3*, and *Y45F10D.4*, which are optimal internal controls for worm qCPR (Hoogewijs *et al.* 2008). Primer sequences were as follows:

*cdc-42* forward: GGTGGCGAGCCATACACATTAGG
*cdc-42* reverse: CTCTCCAACATCCGTTGACACTGG
*pmp-3* forward: TGGTGTCGCGATTACTGTAG
*pmp-3* reverse: GATTTGTTGTCGCAGAGTGG
*Y45F10D.4* forward: CCTGCACAAGTTTGCGTTGCC
*Y45F10D.4* reverse: CGAATCTGCAATTTCATGACATCTCCAC
*hsp-16.48* forward: TCATGCTCCGTTCTCCATTTTCTGATTC
*hsp-16.48* reverse: CTTCTTTGGAGCCTCAATTTGAAGTTTTCC
*hsp-16.1* forward: TTTTGTTCAACGGGCGCTTGC
*hsp-16.1* reverse: GAGGCTCTCCATCTGAATCTTCTGAG
*hsp-70* forward: GAAGGAGAACGTGCTATGACTCG
*hsp-70* reverse: CAGTGATCCATGTTCTTCGAGAGC
*pat-10* forward: GGCCACTCAGATCGGTCAAATCATG
*pat-10* reverse: GTTGTTGATCGGTGAGGTCATCGG.

### Western blotting

Fifty worms were picked into 12.5 μl of nuclease-free water, mixed with an equal volume of 2× Tris-glycine SDS sample buffer (Life Technologies), and frozen at −80°. Following one freeze–thaw cycle, worm samples were boiled, separated on Novex 4–12% Tris-glycine gels (Life Technologies), and transferred to nitrocellulose. Antibodies used for immunoblotting were: 1:1000 anti-β-actin (Sigma) and 1:1000 anti-GFP (Roche).

### Internal ethanol measurements

Ethanol concentrations were measured following a previously published protocol (Alaimo *et al.* 2012). A total of 200 ethanol-treated worms were picked to a tube containing 20 μl of nuclease-free water and frozen at −80°. Worms were thawed, homogenized, and tested for internal ethanol concentration according to the manufacturer’s directions using and NAD-ADH reagent multiple test vial (Sigma). A total of 10 μl of worm homogenate was added to 3 ml of reagent, inverted, and incubated for 10 min. Alcohol concentration was determined by measuring absorbance at 340 nm. Three biological repeats were performed for each strain.

### Behavioral assays

All experiments were performed on young adult hermaphrodite animals from sparsely populated plates grown at 20°. Behavioral experiments were performed in a temperature-controlled room at 20°. Locomotion rate was measured either as thrashing in solution (ethanol experiments) or as body bends on agar plates (ethanol and nicotine experiments) as previously described (Graham *et al.* 2009; Johnson *et al.* 2009; Edwards *et al.* 2012). One thrash was defined as one complete movement from maximum to minimum amplitude and back again. One body bend was defined as one complete sinusoidal movement. For ethanol-sensitivity experiments, single adult hermaphrodites were removed from NGM plates and placed in a Petri dish containing 200 µl of freshly made Dent’s solution (140 mM NaCl, 6 mM KCl, 1 mM CaCl_2_, 1 mM MgCl_2_, and 5 mM HEPES, pH 7.4, with bovine serum albumin at 0.1 mg⋅ml^-1^). Ethanol was previously mixed with Dent’s at the indicated concentrations. For acute ethanol exposure, locomotion (as thrashes per minute) was quantified following a 10-min exposure and normalized as a percentage of the mean thrashing rate of untreated worms measured each day (at least 10 control worms per strain). For nicotine-sensitivity experiments, single adult hermaphrodites were removed from NGM plates and placed on a fresh unseeded NGM plate supplemented with the appropriate nicotine concentration. Locomotion (body bends per minute) was quantified following a 14-min exposure and normalized as a percentage of the mean body bend rate of untreated worms measured each day (at least 10 control worms per strain). For acute ethanol tolerance experiments, we also quantified locomotion as body bends per minute on unseeded NGM plates supplemented with the appropriate ethanol concentration at 10 and 30 min. Locomotion rate here was again normalized to the mean body bend rate of each untreated strain at the indicated times. For heat shock (HS) preconditioning, worms were transferred to seeded, uncrowded NGM plates; the plates were parafilmed and then placed in an incubator at 30° for 1 hr. The plates were then removed to 20° following preconditioning to recover for at least 1 hr before behavioral analysis. Thrashing and body bend rates were quantified within 4 hr post-HS preconditioning. For thermotolerance assays, worms (30 per strain per experiment) were transferred to seeded NGM plates; the plates were parafilmed and then placed in an incubator at 33° for ∼16–18 hr before scoring worms for lethality. All data are expressed as mean ± SE. Significance was tested by Student’s *t*-test, Mann-Whitney *U*-test, or analysis of variance (ANOVA) with Tukey post hoc comparisons as indicated.

### Data availability

Strains are available upon request.

## Results

### HSF-1 is a common modulator of alcohol and nicotine sensitivity

**Figure 1:**
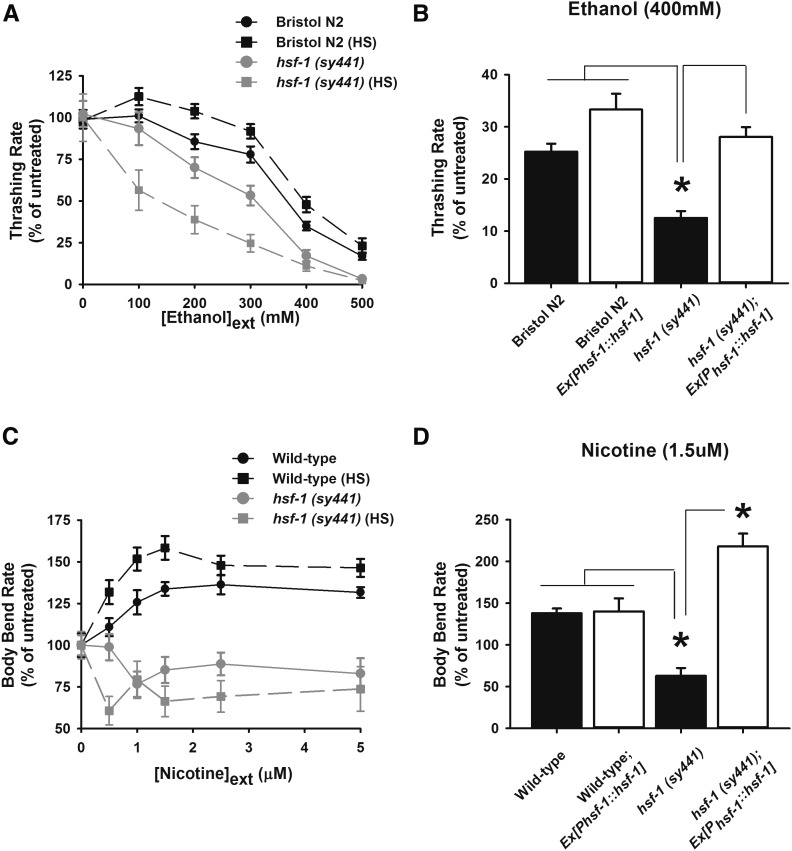
The heat shock transcription factor, HSF-1, is a common modulator of alcohol and nicotine sensitivity. (A) Locomotion rate was determined by quantifying thrashing over a range of external ethanol concentrations after a 10-min exposure. The *hsf-1 sy441* mutant allele increased ethanol sensitivity in comparison to wild type (Bristol N2). Exposure to a preconditioning heat shock (HS) reduced ethanol sensitivity in Bristol N2 worms, but increased ethanol sensitivity further for *hsf-1 sy441* worms. *P* < 0.001 (two-way analysis of variance); *N* = 20 for each strain at each ethanol concentration. (B) Locomotion rate was determined by quantifying thrashing after a 10-min exposure to 400 mM of external ethanol. Transgenic rescue of *hsf-1 (sy441)* worms with wild-type *hsf-1* driven by its endogenous promoter (*hsf-1;Ex[P_hsf-1_*::*hsf-1]*) fully restored ethanol sensitivity to a level statistically equivalent to Bristol N2 wild types. Overexpression of *hsf-1* in Bristol N2 wild type (*wild-type;Ex[P_hsf-1_*::*hsf-1]*) did not alter ethanol sensitivity in comparison to *hsf-1 sy441* or N2 worms. *P < 0.05 (one-way analysis of variance with Tukey post hoc comparisons); *N* = 50 for each strain. (C) Locomotion rate was determined by quantifying body bends over a range of external nicotine concentrations after a 14-min exposure. The *hsf-1 sy441* mutant allele had a reduced nicotine sensitivity in comparison to wild type (Bristol N2). Exposure to a preconditioning HS enhanced nicotine sensitivity in Bristol N2 worms, but had no effect on *hsf-1 sy441* worms. *P < 0.05 (two-way analysis of variance); *N* = 20 for each strain at each ethanol concentration. (D) Locomotion rate was determined by quantifying body bends following a 14-min exposure to 1.5 µM of external nicotine. Transgenic rescue of *hsf-1 (sy441)* worms with *hsf-1* driven by its endogenous promoter (*hsf-1;Ex[P_hsf-1_*::*hsf-1]*) restored the nicotine stimulatory phenotype, significantly increasing sensitivity. Overexpression of *hsf-1* in Bristol N2 (*wild-type;Ex[P_hsf-1_*::*hsf-1]*) did not alter nicotine sensitivity in comparison to Bristol N2. *P < 0.05 (one-way analysis of variance with Tukey post hoc comparisons); *N* = 20 for each strain.

The heat shock transcription factor, HSF-1, is the ubiquitously expressed regulator of stress-activated chaperones (Anckar and Sistonen 2011) contributing to many essential cellular phenotypes. In addition to systemic control of the cellular stress response, HSF-1 is also a critical factor in other complex human diseases, such as cancer, through transcriptional networks distinct from stress (Mendillo *et al.* 2012). We investigated whether HSF-1 could act as a common modulator for physiologically divergent addictive substances. *C. elegans* has a characteristic dose-dependent response to alcohol similar to humans (Davies *et al.* 2003; Graham *et al.* 2009), whereby, as external ethanol concentration is increased, wild-type worms become progressively uncoordinated as quantified by a reduction in locomotion rate (Figure 1A). We tested first for a general role of HSF-1 in determining acute sensitivity to alcohol by examining a mutant *hsf-1* allele (*sy441*) that inhibits its transcriptional activity (Hajdu-Cronin *et al.* 2004). Both the mutant *hsf-1* allele (*sy441*) and RNAi knockdown of *hsf-1* demonstrate two well-established phenotypes of an increase in temperature sensitivity and a decrease in lifespan (Hsu *et al.* 2003; Morley and Morimoto 2004; Prahlad *et al.* 2008; Chiang *et al.* 2012; Kourtis *et al.* 2012; Baird *et al.* 2014). Therefore to preclude any effects of temperature or age, all experiments therefore were performed using young, adult worms at a standardized ambient temperature (20°), unless otherwise indicated. To rule out the possibility that the *hsf-1sy441* mutation affected nematode permeability or ethanol metabolism, we measured internal concentrations of animals exposed to high, 400 mM external ethanol and found similar internal concentrations in both wild-type Bristol N2 (70.8 ± 10.2 mM) and *hsf-1sy441* worms (59.8 ± 8.1 mM) at levels comparable with previously published concentrations (Alaimo *et al.* 2012). Finally, to minimize the influence of small differences in basal locomotion, locomotion of drug-treated animals was quantified normalized to untreated worms of the same strain as done previously (Davies *et al.* 2003; Graham *et al.* 2009; Johnson *et al.* 2013); however, basal rates for all strains can be found in Table S1.

In comparison to wild-type worms, *hsf-1sy441* showed a significant enhancement in the depressive effects of ethanol on nematode locomotion (Figure 1A) that could be restored to a statistically equivalent level to Bristol N2 upon transgenic rescue of wild-type *hsf-1* under its endogenous promoter (Figure 1B). Overexpression of wild-type *hsf-1* under its endogenous promoter in a wild-type (Bristol N2) background had no significant additional effect to ethanol sensitivity. Finally, like mammals, nematodes display acute tolerance to alcohol (Davies *et al.* 2004) and we determined that this neuroadaptive response was also absent in *hsf-1sy441* mutants and could be re-established by transgenic rescue with wild-type *hsf-1* under its endogenous promoter (Figure S1).

Exposure to heat shock (HS) classically upregulates the expression of many HSPs downstream of the HSF-1 transcription factor including members of the HSP-70 and HSP-16 family of proteins (Hsu *et al.* 2003; Larance *et al.* 2011; Chiang *et al.* 2012; Kourtis *et al.* 2012; Baird *et al.* 2014) and induces resistance to a subsequent temperature stress (Parsell and Lindquist 1993; Morimoto 2011; Kourtis *et al.* 2012). We exposed worms to a preconditioning HS protocol (30° for 1 hr followed by 1 hr of recovery) and confirmed an induction of an HSF-1-dependent temperature stress resistance for both Bristol N2 controls and transgenic rescues of *hsf-1sy441*, but not the *hsf-1sy441* mutants themselves (Figure S2). We next investigated whether a preconditioning HS would have the opposite effect on alcohol sensitivity as the *hsf-1sy441* mutation. As predicted, HS preconditioned Bristol N2 worms showed a reduction in the depressive effects of alcohol over the tested range of concentrations, whereas the preconditioning HS actually enhanced the ethanol sensitivity for the *hsf-1sy441* mutants (Figure 1A).

Following these results, we tested for a role for HSF-1 in acute sensitivity to nicotine. Wild-type *C. elegans* has an acute dose-dependent response to nicotine at concentrations similar to humans (Feng *et al.* 2006). Exposure of Bristol N2 worms to a preconditioning HS enhanced the stimulatory effects of nicotine, whereas in the *hsf-1sy441* mutants, nicotine instead acted as a depressant (Figure 1C). As seen with alcohol, the preconditioning HS actually enhanced this reversed, depressive nicotine phenotype for the *hsf-1sy441* mutants. Transgenic rescue of *hsf-1sy441* with wild-type *hsf-1* under its endogenous promoter again restored and, in fact, significantly increased the stimulatory phenotype of nicotine (Figure 1D). Overexpression of wild-type *hsf-1* under its endogenous promoter in a wild-type (Bristol N2) background again had no significant additional effect to nicotine sensitivity. From these results we conclude that HSF-1 is a novel common modulator of substance sensitivity.

### Modulation of drug sensitivity requires neuronal HSF-1 expression

**Figure 2:**
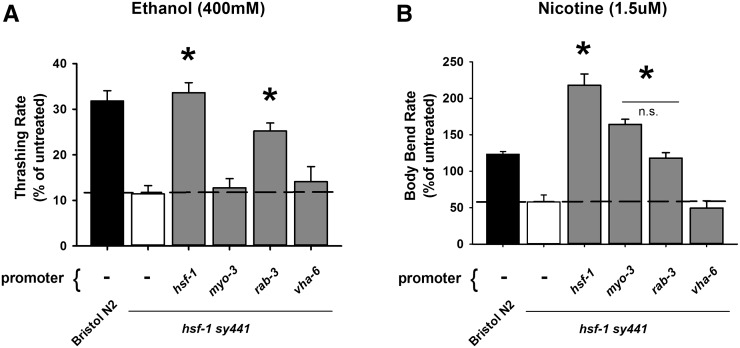
Modulation of drug sensitivity requires neuronal HSF-1 expression. (A) In comparison to *hsf-1 sy441*, transgenic rescue with either the *hsf-1* promoter (*P_hsf-1_*) or a panneuronal (*P_rab-3_*) promoter restored ethanol sensitivity equal to Bristol N2. Expression in muscle (*P_myo-3_*) or the intestine (*P_vha-6_*) did not rescue the *hsf-1 (sy441)* phenotype. *P < 0.05 (one-way analysis of variance with Tukey post hoc comparisons); *N* = 30 for each strain. (B) In comparison to *hsf-1 sy441*, transgenic rescue with the panneuronal (*P_rab-3_*) or the body-wall muscle (*P_myo-3_*) promoters restored nicotine concentration statistically equivalent to Bristol N2, whereas rescue with the *hsf-1* (*P_hsf-1_*) promoter increased nicotine sensitivity further. Expression in the intestine (*P_vha-6_*) had no effect. n.s., nonsignificant. *P < 0.05 (one-way analysis of variance with Tukey post hoc comparisons); *N* = 20 for each strain.

The alteration in locomotor speeds following ethanol or nicotine could reflect changes in the coordinated patterns of motor activity generated by the worms’ CNS or an effect on the strength of the end-point signal at the body wall neuromuscular junction. We and others have demonstrated that the effects of ethanol are independent of signaling strength and basal locomotion rates (Davies *et al.* 2003; Graham *et al.* 2009); however, nicotine directly activates the postsynaptic muscle and HSF-1 is a transcription factor expressed ubiquitously in all cell types. Therefore we wanted to determine the localization of HSF-1 required for rescue of both the acute alcohol and nicotine phenotypes by tissue-specific transgenic expression. Both transgenic rescue of *hsf-1* under its endogenous promoter (*hsf-1;Ex[P_hsf-1_*::*hsf-1]*) or using a panneuronal promoter (*hsf-1;Ex[P_rab-3_*::*hsf-1]*) rescued the alcohol phenotype equally in *hsf-1sy441* mutants, whereas transgenic expression using a body-wall muscle promoter (*hsf-1;Ex[P_myo-3_*::*hsf-1]*) was insufficient (Figure 2A). To test a role for detoxification, we also verified that transgenic expression using an intestinal promoter (*hsf-1;Ex[P_vha-6_*::*hsf-1]*) was insufficient to rescue the ethanol phenotype. Similar results for the nicotine phenotype of *hsf-1sy441* mutants was achieved by transgenic expression of *hsf-1* under a panneuronal promoter (*hsf-1;Ex[P_rab-3_*::*hsf-1]*), but also a body-wall muscle promoter (*hsf-1;Ex[P_myo-3_*::*hsf-1]*) in comparison to rescue with the endogenous promoter (*hsf-1;Ex[P_hsf-1_*::*hsf-1]*) (Figure 2B). A requirement for HSF-1 expression in muscle for nicotine sensitivity is perhaps unsurprising as nAChRs are expressed in both muscle and nervous system in *C. elegans* (Jones and Sattelle 2004). Similar to alcohol, there was no effect when transgenically expressing with the intestinal promoter (*hsf-1;Ex[P_vha-6_*::*hsf-1]*). From these results we conclude that neural expression of HSF-1 is sufficient for modulation of substance sensitivity.

### HSP-16.48 acts downstream of HSF-1

HSF-1 functions as a transcription factor that regulates the expression of a host of downstream targets, in both the stressed and unstressed conditions. Control of HSP expression is an integral component of either condition. HSPs are ubiquitous proteins that function to block protein aggregration, assist in protein folding or refolding, facilitate protein degradation, and stabilize protein–protein interactions (Kampinga and Craig 2010). We characterized whether constitutively expressed HSPs were involved in substance sensitivity downstream of HSF-1 by performing a broad RNAi screen of HSPs in the *C. elegans* genome and comparing with RNAi of *hsf-1* and genes previously associated with alcohol (*rab-3* and *slo-1*) (Davies *et al.* 2003; Kapfhamer *et al.* 2008) or nicotine (*trp-2* and *acr-15*) (Feng *et al.* 2006) sensitivity in worms (Figure S3). The RNAi screen was performed by feeding (Kamath and Ahringer 2003; Kamath *et al.* 2003) using the *rrf-3pk1426* strain, as it has been widely characterized and has enhanced efficiency in neurons (Zhuang and Hunter 2011), and then quantifying the locomotion rate of alcohol- or nicotine-treated animals as a percentage of untreated responses.

**Figure 3:**
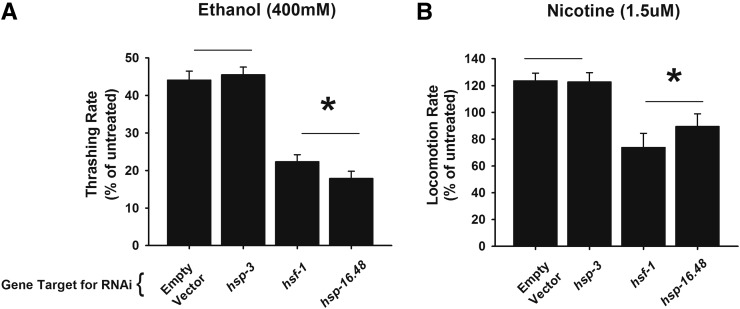
RNAi of HSP-16.48 phenocopies HSF-1 in the modulation of drug sensitivity. (A) RNAi knockdown of the small HSP, *hsp-16.48*, statistically phenocopied the ethanol sensitivity phenotype of *hsf-1* RNAi knockdown in comparison to empty vector control or knockdown of the endoplasmic reticulum chaperone *hsp-3*. Note that *hsp-16.48/hsp-16.49* is a gene duplication and, as such, RNAi should affect expression of both genes. *P < 0.05 (one-way analysis of variance with Tukey post hoc comparisons); *N* = 20 for each RNAi target. (B) RNAi knockdown of the small HSP, *hsp-16.48*, phenocopied the nicotine sensitivity phenotype of *hsf-1* RNAi knockdown in comparison to empty vector control or knockdown of the endoplasmic reticulum chaperone *hsp-3*. *P < 0.05 (one-way analysis of variance with Tukey post hoc comparisons); *N* = 20 for each RNAi target.

We first determined that knockdown of *hsf-1* would have similar effects on drug sensitivity as did the *hsf-1sy441* mutant. In comparison to the empty feeding vector control, RNAi of *hsf-1* indeed altered both ethanol and nicotine sensitivity as predicted (Figure S3). RNAi of the additional positive controls, *rab-3* and *slo-1*, reduced ethanol sensitivity as has been shown for *loss-of-function* mutants of these genes (Davies *et al.* 2003; Kapfhamer *et al.* 2008). RNAi of the additional positive controls, *trp-2* and *acr-15*, reduced nicotine sensitivity as has been shown for *loss-of-function* mutants of these genes (Feng *et al.* 2006). We were next interested in identifying those HSP genes that affected sensitivity to both drugs to a similar level to *hsf-1*. For this we adopted a threshold for gene RNAi that was within 5% or greater of the *hsf-1* RNAi phenotype. Although a number of RNAi targets matched our threshold conditions for either ethanol (*e.g.*, *hsp-16.41*) or nicotine (*e.g.*, *F11F1.1*), specifically, they did not affect sensitivity to the other drug. We found that RNAi of only one gene target, *hsp-16.48*, was able to phenocopy *hsf-1* RNAi for both alcohol and nicotine sensitivity. We therefore concentrated our efforts on HSP-16.48 acting downstream of HSF-1 in both phenotypes and repeated the RNAi experiments, statistically verifying that, in comparison to either the empty vector control or RNAi or *hsp-3*, RNAi of both *hsf-1* and *hsp-16.48* significantly reduced alcohol and nicotine sensitivity to an equivalent level (Figure 3, A and B).

**Figure 4:**
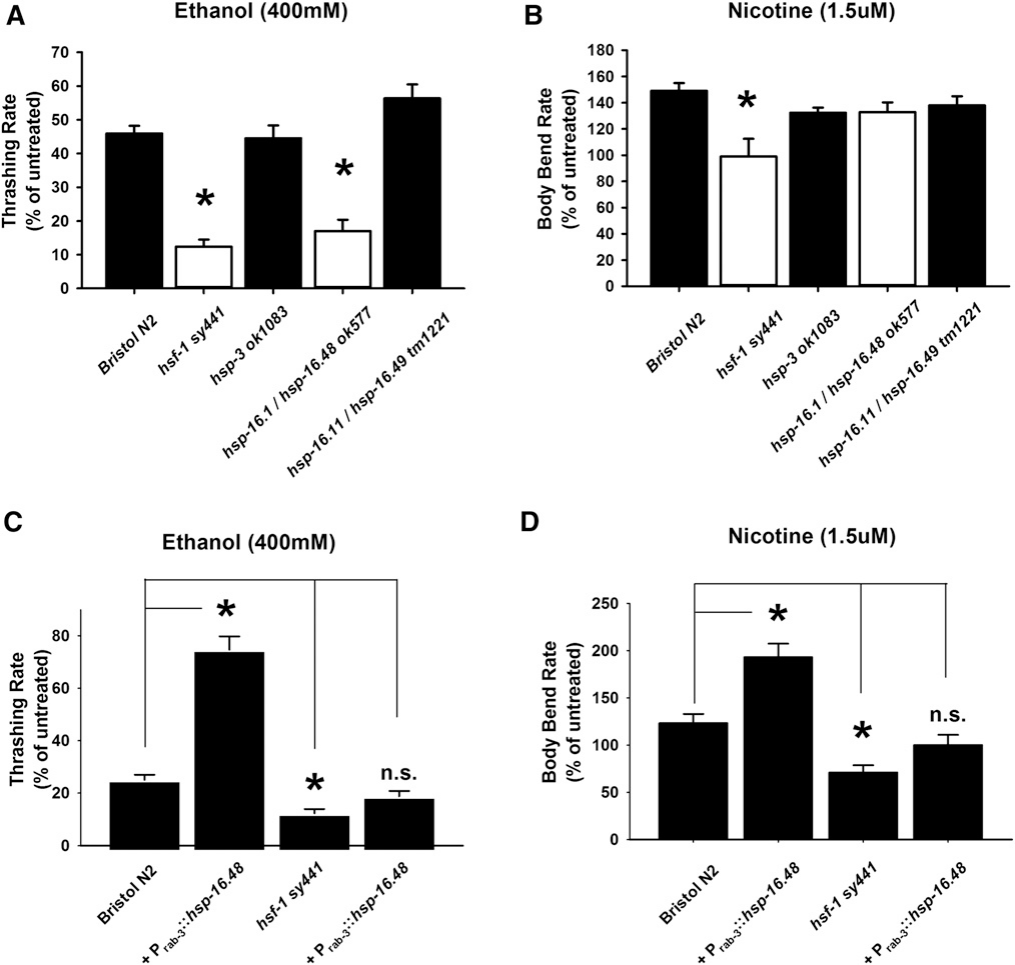
Neuronal HSP-16.48 overexpression alters drug sensitivity in both N2 and *hsf-1 sy441*. *Loss-of-function* mutations of individual heat shock protein (HSP) genes were analyzed. Note that, due to genetic proximity, *hsp-16.1/hsp-16.48* and *hsp-16.11/hsp-16.49* mutant alleles affect two heat shock protein genes. (A) In comparison to Bristol N2 and *hsf-1 sy441* worms, the ethanol phenotype of *hsf-1 sy441* was phenocopied by the *hsp-16.1/hsp-16.48 ok577* mutant. *P < 0.05 (analysis of variance with Tukey post hoc comparisons); *N* = 10 for each strain. (B) In comparison to Bristol N2 and *hsf-1 sy441* worms, the nicotine phenotype of *hsf-1 sy441* was not phenocopied by either the *hsp-16.1/hsp-16.48 ok577* or the *hsp-16.11/hsp-16.49 tm1221* mutants. *N* = 20 for each strain. (C) Panneuronal expression of *hsp-16.48* is sufficient to restore ethanol sensitivity of *hsf-1 sy441* worms to a statistically equivalent level to Bristol N2 worms. n.s., nonsignificant. Panneuronal expression of *hsp-16.48* reduces ethanol sensitivity of Bristol N2 worms to a significantly greater level than for *hsf-1 sy441* worms. *P < 0.05 (one-way analysis of variance with Tukey post hoc comparisons); *N* = 30 for each. (E) Panneuronal expression of *hsp-16.48* is sufficient to restore nicotine sensitivity of *hsf-1 sy441* worms to a statistically equivalent level to Bristol N2 worms. Panneuronal expression of *hsp-16.48* increases nicotine sensitivity to a significantly greater level than for *hsf-1 sy441* worms. *P < 0.05 (one-way analysis of variance with Tukey post hoc comparisons); *N* = 20 for each strain.

HSP-16.48 is a small heat-shock protein most closely homologous to *C. elegans* HSP-16.1 and othologous to human α-crystallin (Figure S4), a ubiquitously expressed protein associated with cataract and a number of neurodegenerative diseases and cancer (Arrigo *et al.* 2007). We next attempted to replicate the results of the RNAi screen by testing individual mutants of *hsp-16.48* (the *ok577* allele) and comparing with the *hsf-1sy441* mutant (Figure 4, A and B). Consistent with the RNAi results, a mutant of the *hsp-3* (*ok1083*) gene had no effect on either sensitivity to alcohol or nicotine. In comparison to Bristol N2 wild types, however, we found that the *hsp-16.48ok577* mutant only phenocopied *hsf-1sy441* for alcohol sensitivity (Figure 4, A and B). We predict that this is most probably the consequence of two factors. First, the *hsp-16.48ok577* allele also deletes the closely related small HSP, *hsp-16.1*, which may have a combinatorial effect with *hsp-16.48*, although *hsp-16.1* RNAi did not alter sensitivity to ethanol or nicotine. A more likely explanation is that *hsp-16.48*, as well as *hsp-16.1*, has undergone evolutionary genetic duplication resulting in the identical *hsp-16.49* and *hsp-16.11* genes, respectively (Figure S5). We also tested the mutant allele of *hsp-16.49* and *hsp-16.11* (*tm1221*), but found that this too was phenotypically similar to Bristol N2s. Given this genetic duplication, a lack of complete phenocopy is perhaps unsurprising. Although RNAi will knockdown both *hsp-16.48* and *hsp-16.49*, the mutant allele only deletes one of two copies.

To support further the hypothesis that *hsp-16.48* was indeed acting downstream of *hsf-1* in both alcohol and nicotine sensitivity, we tested whether transgenic overexpression of *hsp-16.48* alone could alter sensitivity to both alcohol and nicotine. We found that neuronal expression of *hsp-16.48* in Bristol N2 wild-type worms indeed had the opposite effect to the *hsf-1sy441* phenotype (Figure 4, C and D). We also determined that overexpression of *hsp-16.48* alone could partially rescue the *hsf-1sy441* phenotype, being statistically equivalent to wild-type drug sensitivity, but significantly lower than the *hsp-16.48* overexpression in wild-type worms. Finally, despite the very high sequence similarity (Figure S4), transgenic overexpression of closely homologous *hsp-16.1* or an unrelated HSP-70 chaperone, *hsp-1*, did not phenocopy *hsp-16.48* overexpression (Figure S6). Taken together, these results indicate that, although HSP-16.48 overexpression alone is not sufficient to compensate completely for a lack of functional HSF-1 in the assayed phenotypes, the HSF-1-dependent effects on drug sensitivity require HSP-16.48 and that HSP-16.48 expression alone is sufficient to alter both alcohol and nicotine sensitivity.

### HSP-16.48 functions independently of the heat shock stress response

Exposure of cells, tissues, or whole organisms to elevated temperatures activates the HSF1-dependent HS stress response (Anckar and Sistonen 2011). During this response, the HSF-1 transcription factor is activated, stimulating a large increase in expression of many genes. The classically defined genes that are overexpressed in response to temperature stress are the HSPs (Hsu *et al.* 2003; Prahlad *et al.* 2008; Larance *et al.* 2011; Kourtis *et al.* 2012), such as the α-crystallins, although expression of other genes can also be affected. The upregulated HSPs are primarily characterized to function as cellular chaperones, binding misfolded or aggregation prone proteins to increase survival during times of cellular stress. Alternatively, recent studies have shown that HSF-1 can regulate transcriptional programs independent of cellular stress (Page *et al.* 2006; Mendillo *et al.* 2012) and that α-crystallins themselves can function through interactions with correctly folded clients (Delbecq and Klevit 2013). Despite a lack of correlation of drug sensitivity with many of the heat shock protein chaperones, previous work has shown that exposure of nematodes to alcohol at high, unphysiological (1.2 M) concentrations does indeed induce the heat shock response in *C. elegans* (Kwon *et al.* 2004). We were interested to determine whether exposure to ethanol or nicotine at the concentrations used in our experiments was simply activating the heat shock transcription pathway and upregulating levels of HSP-16.48.

**Figure 5:**
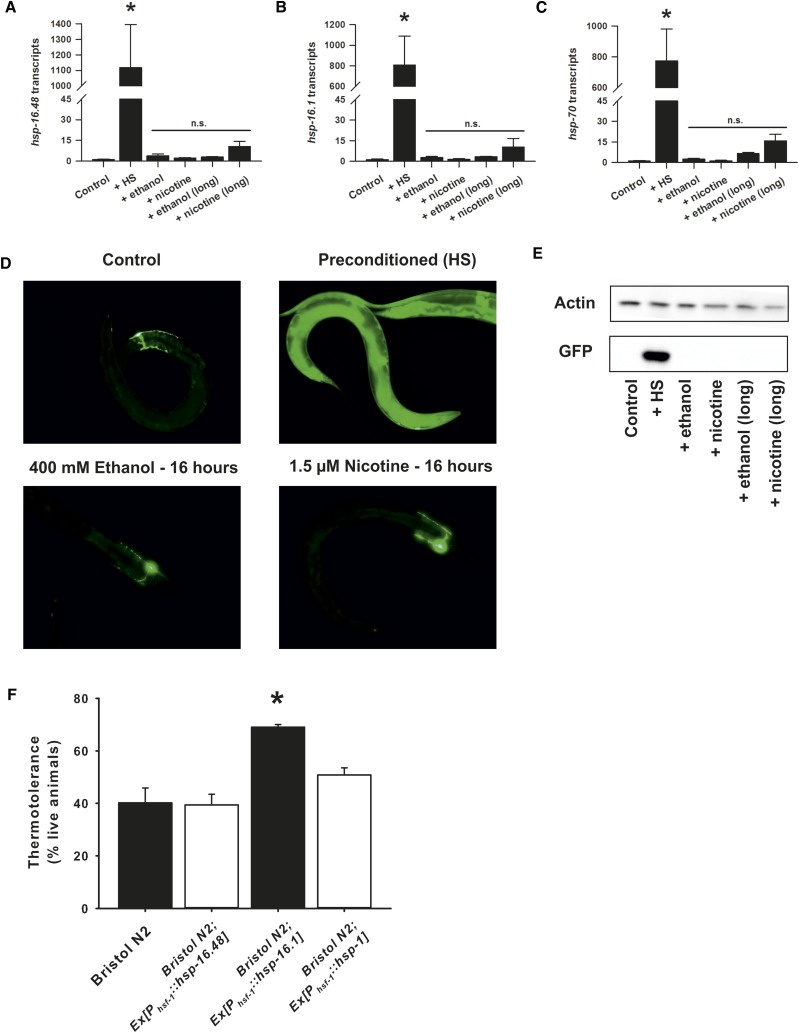
The function of HSP-16.48 in drug sensitivity is unrelated to the heat shock stress response. Exposure to ethanol or nicotine do not activate the HS stress pathway at the concentrations used in this study. Quantitative PCR of *hsp-16.48* (A), *hsp-16.1* (B), and *hsp-70* (C) shows an upregulation in transcript expression in response to heat shock (+HS), but not to ethanol or nicotine exposure. There was also no effect on transcript expression in response to a longer ethanol or nicotine exposure that matched the HS protocol (1-hr exposure, 1-hr recovery). (D) Photographs of worms expressing green fluorescent protein (GFP) under the control of the *hsp-16.48* promoter. (Top left) GFP expression was visualized at low levels without heat shock preconditioning. The cell that most reliably expressed GFP at visual levels under basal conditions was preliminarily identified as the DB2 motorneuron (indicated by arrow). (Top right) A large increase in GFP expression occurred in most cells following exposure to our heat shock protocol, indicating the activation of the heat shock stress response. Exposure of worms to the concentrations of alcohol (bottom left) or nicotine (bottom right) used in this study did not increase GFP expression, even after long-term (16 hr) exposure. Bar, 0.1 mm. (E) Western blot of *Phsp-16.48*::GFP. Protein expression is upregulated in response to HS, but not acute ethanol or nicotine exposure or longer exposure matching the HS protocol. (F) Transgenic overexpression in Bristol N2 worms of *hsp-16.1*, but not *hsp-16.48* or the HSP-70 chaperone *hsp-1*, induces resistance to a subsequent temperature stress. Surviving worms of the indicated strains were quantified following exposure to 33° for 16–18 hr. In comparison to Bristol N2 worms, only Bristol N2;*Ex[P_hsf-1_*::*hsp-16.1]* demonstrated an improvement in survival. *P < 0.05 (one-way analysis of variance with Tukey post hoc comparisons); *N* = 3 (of 30 animals each per strain).

Using quantitative PCR, we verified that exposure of *C. elegans* to a 30° heat shock indeed induced a massive upregulation in expression of various heat shock proteins, including *hsp-16.48* (Figure 5A), *hsp-16.1* (Figure 5B), and *hsp-70* (Figure 5C). In response to the ethanol or nicotine concentrations used in our experiments, however, there was no change in messenger RNA (mRNA) expression of any of these genes. Both nicotine and ethanol are poisons and it remained possible that the exposure at these concentrations was inducing a HS response that was unable to be quantified within the short time frame of the experiment. We therefore measured expression in response to a longer drug exposure protocol that matched our HS (1-hr exposure followed by 1-hr recovery). Again, neither nicotine nor ethanol had any significant effects on heat shock protein expression (Figure 5, A–C). We also constructed a reporter strain that expressed GFP under the control of the *hsp-16.48* promoter (Figure 5D). GFP was expressed sporadically in a variety of cells, including neurons, at low levels and reliably expressed in some head neurons at a higher level. As expected, expression of GFP under the control of the heat shock promoter was increased substantially and in most cells of the worm in response to the heat shock protocol (Figure 5D). In agreement with the qPCR experiments, exposure to either exogenous ethanol or nicotine did not increase expression of GFP regardless of the length of exposure (Figure 5D). Higher magnification images of the nematodes demonstrated that there were no obvious tissue-specific alterations in GFP expression (Figure S7). Finally the extent of GFP expression in response to temperature stress, ethanol, or nicotine were quantified by Western blot, emphasizing the inability of ethanol or nicotine at these concentrations to activate the classical HS stress response (Figure 5E).

Although our experimental conditions were not enough to induce the HS stress response, it remained possible that the endogenous levels of *hsp-16.48* were acting as chaperones to protect cells from a low level of stress that was insufficient to activate upregulation in heat shock protein expression. Indeed the highly similar small HSP, *hsp-16.1*, is upregulated in response to heat shock (Figure 5B) and acts at endogenous levels as a cellular chaperone to protect against heat-induced neurodegeneration (Kourtis *et al.* 2012); yet, RNAi of *hsp-16.1* here did not phenocopy RNAi of *hsf-1* or *hsp-16.48* in sensitivity to addictive substances, and overexpression of *hsp-16.1* was not beneficial to drug sensitivity. Given their structural similarities, we tested whether overexpression of HSP-16.48 could act as a chaperone under specific environmental conditions and protect against a temperature stress. As described previously (Kourtis *et al.* 2012), we found that overexpression of *hsp-16.1* indeed protected animals from a temperature stress; however, overexpression of neither *hsp-16.48* nor *hsp-1* had any protective effect (Figure 5B), despite the ability of *hsp-16.48* overexpression to alter both ethanol and nicotine sensitivity. These contrasting data between overexpression of *hsp-16.48* and *hsp-16.1* argue strongly that the very small variations in protein sequence determine the phenotypic differences in protein function, either as a classical temperature stress-dependent protection of cells or as a novel regulation of sensitivity to addictive substances.

### Drug sensitivity effects appear to be independent of *pat-10*

In addition to increasing HSP expression hundreds-fold, HS can also affect the expression of other genes. One such gene encodes the calcium binding protein PAT-10 whose expression is upregulated twofold in response to HS (Baird *et al.* 2014). Recently, *pat-10* has been shown to be involved in thermotolerance and lifespan increases as a consequence of *hsf-1* overexpression through a protection of cytoskeletal integrity (Baird *et al.* 2014). We investigated a potential role of *pat-10* in determining our drug sensitivity phenotypes. Although *pat-10* expression was increased in response to heat shock, it was unaffected by ethanol or nicotine exposure regardless of the length of exposure (Figure S8). Second, we visualized basic aspects of muscle integrity in response to ethanol or nicotine exposure. Upon HS, muscle filaments become disorganized and this is thought to be a contributing factor to thermotolerance (Baird *et al.* 2014). We confirmed that the muscle filaments became disorganized in response to heat shock, but could see no overt effects of ethanol or nicotine exposure (Figure S8). Using a GFP-tagged synaptobrevin/VAMP marker for synapses, we could also find no evidence for overt effects of ethanol or nicotine on neuronal morphology (Figure S8). Finally, overexpression of *pat-10* has been previously demonstrated to increase thermotolerance and lifespan of nematodes (Baird *et al.* 2014). We analyzed this *pat-10* overexpression strain and found that it had no effect on sensitivity to ethanol in our assays (normalized Bristol N2 thrashing rate = 25.14 ± 1.90; normalized *pat-10* overexpression thrashing rate = 25.16 ± 3.24; *N* = 30 for each strain). Therefore, our preliminary evidence suggests that the pharmacological effects of these drugs at these experimental conditions are independent of *pat-10*.

### Structure–function analysis indicates a seven amino acid domain underlying the drug sensitivity phenotype of HSP-16.48

**Figure 6:**
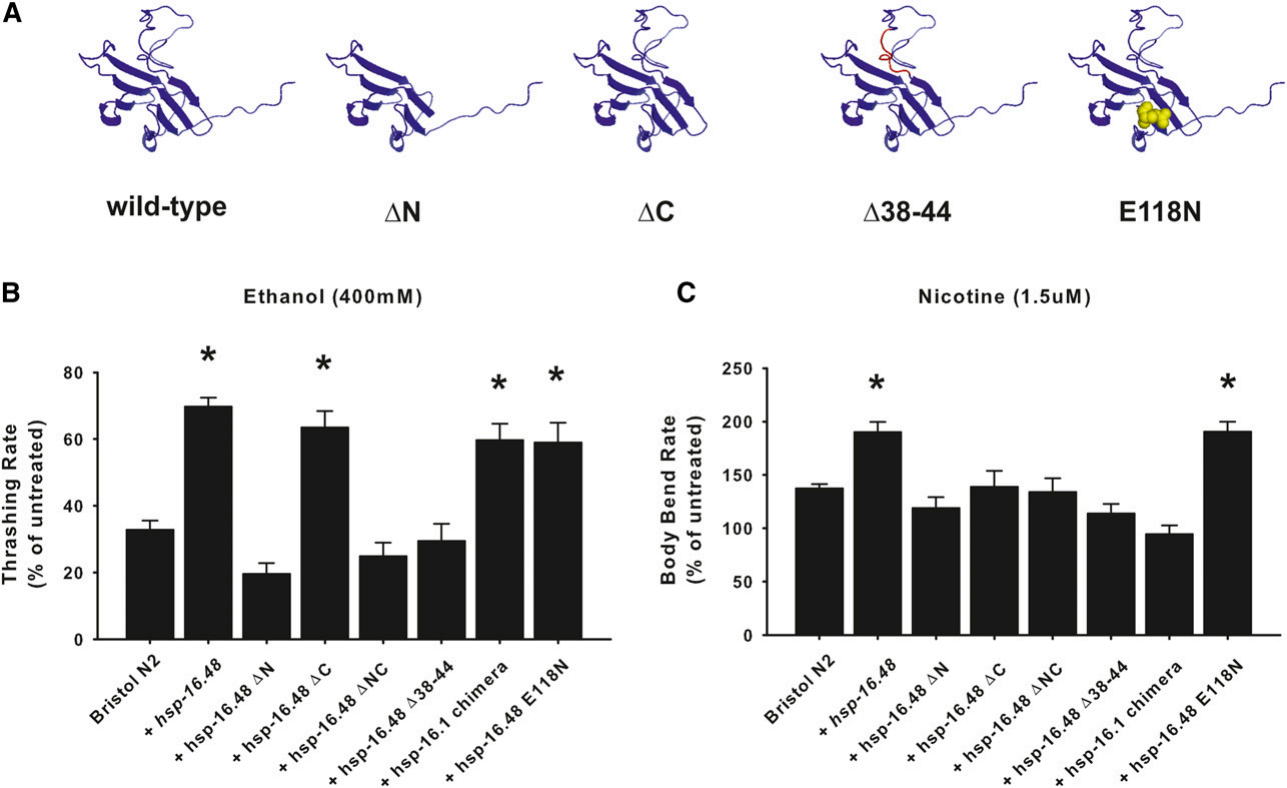
HSP-16.48 function requires an essential seven amino acid section of its N-terminal domain. (A) Schematic demonstrating the truncation or mutations used in structure–function analysis of HSP-16.48. The position of the seven amino acids (Δ38–44) in the deletion mutant are indicated in red. The position of the Glu118 amino acid is indicated in yellow. (B) The function of HSP-16.48 in ethanol sensitivity is determined by the inclusion of amino acids 36–44 within its N terminus. In comparison to Bristol N2 worms, truncation of either the N terminus (ΔN), both the N and C termini (ΔNC), or the seven amino acids (Δ38–44) only block the effects of HSP-16.48 overexpression on ethanol sensitivity. Fusing the N terminus of HSP-16.48 to the crystallin domain and C terminus of HSP-16.1 (HSP-16.1 chimera) converts the phenotype of HSP-16.1 into HSP-16.48. An E118N mutation that blocks oligomerization of crystallin proteins has no effect on HSP-16.48 function. *P < 0.05 (one-way analysis of variance with Tukey post hoc comparisons); *N* = 30 for each strain. (C) The function of HSP-16.48 in nicotine sensitivity is determined by both amino acids 36–44 within the N terminus plus its C-terminal domain. In comparison to Bristol N2 worms, truncation of the N terminus (ΔN), the C terminus (ΔC), both the N and C termini (ΔNC), or seven amino acids (Δ38–44) only block the effects of HSP-16.48 overexpression on nicotine sensitivity. The HSP-16.1 chimera could not convert the phenotype of HSP-16.1 into HSP-16.48. The E118N mutation had no effect on HSP-16.48 function. *P < 0.05 (one-way analysis of variance with Tukey post hoc comparisons); *N* = 35 for each strain.

Both *hsp-16.1* and *hsp-16.48* are expressed in most cells of *C. elegans* and their expression is upregulated in response to stress; yet, they appear to have completely separate functions. Given the similarity of protein structure, we were interested in identifying the section of the protein that determined this drug sensitivity phenotype of HSP-16.48. Structurally, all small HSPs comprise a central crystallin domain with N- and C-terminal domains of variable length (Figure 6A and Figure S4). The distinction in phenotype between *hsp-16.1* and *hsp-16.48* indicates that the inclusion of a crystallin domain does not necessarily predetermine protein function. We first constructed and analyzed the phenotypic effects of a number of truncation mutations (ΔN, ΔC, and ΔNC) of HSP-16.48 (Figure 6, B and C). In comparison to Bristol N2 wild types, the phenotypic effects of panneuronal HSP-16.48 overexpression on both ethanol and nicotine sensitivity were eliminated when the N terminus (+*hsp-16.48 ΔN*) was truncated (Figure 6, B and C). In contrast, expression of a C-terminal truncation (*+hsp-16.48 ΔC*) only eliminated the nicotine sensitivity. Closer examination of the N-terminal sequences of HSP-16.1 and HSP-16.48 identified a short stretch of seven amino acids that distinguished the HSP-16.48 sequence (Figure S4). Neuronal expression of a mutant protein with only these seven amino acids removed (*+hsp-16.48 Δ38-44*) was phenotypically indistinguishable to the full N-terminal deletion, showing a complete abolition of the effects of HSP-16.48 overexpression on alcohol and nicotine sensitivity (Figure 6, B and C). Next we determined whether we could convert the phenotype of HSP-16.1 in ethanol sensitivity (Figure S6) into HSP-16.48 by switching only their N-terminal regions (*+hsp-16.1 chimera*). Consistent with the C-terminal deletion results, expression of the HSP-16.1 chimera only affected the ethanol sensitivity effects (Figure 6, B and C).

These data indicate that the α-crystallin-like protein, HSP-16.48, functions through its N and C termini rather than through a classical chaperone activity that occurs during stress. It could be argued, however, that the N- and C-terminal truncations may have had indirect effects on crystallin domain oligomerization. We therefore verified that the function of HSP-16.48 was completely independent of its chaperone function by introducing an E118N point mutation that acts as a dominant-negative inhibitor of human α-crystallin (Liu *et al.* 2006). Neuronal expression of this mutation (*+hsp-16.48 E118N*) altered ethanol and nicotine sensitivity to equivalent levels as overexpression of HSP-16.48 (Figure 6, B and C). As overexpression of none of the mutant versions of HSP-16.48 had an effect similar to *hsp-16.48* RNAi, we concluded that none of the mutants were acting in a dominant-negative fashion. The results instead argued that the mutants that had no effect were nonfunctional in drug sensitivity.

## Discussion

Substance addiction is a complex disease with a large number of genetic influences. Although great insight into addiction has come from multiple recent GWAS studies, very few candidates have been reliably and consistently identified (Bierut *et al.* 2007; Edenberg *et al.* 2010; Bierut 2011; Morozova *et al.* 2012). For both alcohol and nicotine, a number of genes have been recognized as being statistically significant, such as the well-documented alcohol dehydrogenase and the nicotinic acetylcholine receptors; however, the contribution from identified genes are estimated to contribute only a fraction of the heritability (Bierut 2011; Wang *et al.* 2012; Demers *et al.* 2014). Additional complexity for genetic factors of addiction in general derives from the discrete targets for the individual addictive substances within the nervous system, making common determinants inherently difficult to identify. In this study, we establish a novel genetic pathway underlying the dose-dependent effects of diverse, exogenous addictive substances within the nervous system. Involvement of the HSF-1 transcription factor and its downstream gene targets could permit broad phenotypic effects through changes in gene expression programs, both in direct response to addictive substances and also in the determination of their efficacy. The downstream identification of a specific α-crystallin protein as a common determinant of drug sensitivity highlights it as a potential target for pharmacological intervention. The finding that the C terminus of HSP-16.48 only is dispensable for alcohol sensitivity, whereas both the N and C termini are required for nicotine, implies that the commonality of this genetic pathway for drug sensitivity ends at the level of the HSP-16.48 protein.

HSF-1 is the heat shock transcription factor that drives chaperone overexpression in response to cellular stress. Both RNAi of *hsf-1* and the *hsf-1sy441* allele display various stress-dependent phenotypes, including increased temperature sensitivity, an absence of the heat shock-induced decrease in temperature sensitivity, and a shortened lifespan (Hsu *et al.* 2003; Morley and Morimoto 2004; Prahlad *et al.* 2008; Chiang *et al.* 2012; Kourtis *et al.* 2012; Baird *et al.* 2014). Are the *hsf-1sy441* mutants simply overly sensitive to any impairment? A clear delineation between pharmacological effects and stress-dependent effects is inherently difficult, given the importance of HSF-1 as both a stress-dependent and stress-independent transcription factor. Our data, however, demonstrate that both RNAi of *hsf-1* and the *hsf-1sy441* mutants show increased sensitivity to ethanol over a range of concentrations, whereas they are not more sensitive to nicotine. The dose-response curve indicates that the effect of nicotine on locomotion rates is both altered from wild type and fairly constant over a range on concentrations. In addition, we show that various mutants (*hsp-16.48ok577*) and transgenics (*N2 + hsp-16.48ΔC*) have altered ethanol responses, but no difference in nicotine sensitivity. These data clearly demonstrate that the pharmacological effects of nicotine and ethanol can be specific, depending on the genetic background. Finally, we show that the small heat shock protein *hsp-16.48* acts downstream of *hsf-1* in ethanol and nicotine; yet, is independent of impairment due to temperature stress. The closely related *hsp-16.1* conversely does not act downstream of *hsf-1* in ethanol and nicotine, but does act downstream of *hsf-1* in temperature-dependent neurodegeneration (Kourtis *et al.* 2012). It appears therefore that the presented evidence supports the hypothesis that drug sensitivity is distinct from the HSF-1-dependent stress phenotype and does not simply represent a broad increase in sensitivity to any general impairment.

We present two additional novel phenotypes for *hsf-1* RNAi and the *hsf-1sy441* mutant allele—an increase in ethanol sensitivity and an absence or reversal of nicotine sensitivity. Ethanol and nicotine are both pharmacological toxins and exposure to sufficiently high levels can activate the HS stress response (Kwon *et al.* 2004). Are the effects seen here simply a result of the activation of the heat shock response to cellular stress? A definitive answer may still require further experimentation; however, we present three lines of evidence that suggest a role independent of cellular stress. First, there was a complete absence of a quantifiable HS stress response following drug exposure either at the level of mRNA or protein expression. This was evident both as a direct response to the drug or where we applied a longer exposure with recovery time similar to HS. While this does not preclude a HS-independent cellular stress response, it does demonstrate that the application of ethanol or nicotine at these concentrations do not activate the HS stress response. Second, overexpression of other protective chaperones in general and indeed the very similar small HSP, HSP-16.1, could not affect drug sensitivity. Structure–function analysis indicated that simply overexpressing a crystallin domain had no effect on drug sensitivity. Additionally, removal of only seven amino acids from the N terminus blocked the effects of HSP-16.48 on drug sensitivity. While we are unable to prove categorically that the HSP-16.48 Δ38-44 retains chaperone activity (or indeed that wild-type HSP-16.48 has any endogenous chaperone activity), sequence analysis indicates that this mutant should be extremely similar to HSP-16.1 which does act as a chaperone during temperature-dependent neurodegeneration (Kourtis *et al.* 2012). Finally, we show that the introduction of a point mutation that is known to inhibit the chaperone function of other crystallin proteins did not curtail the positive phenotype of HSP-16.48 overexpression.

Small HSPs are thought to function solely as molecular chaperones, stabilizing proteins and preventing irreversible aggregation (Garrido *et al.* 2012). If our evidence points to a stress-independent basis for HSP-16.48 function, what precisely is HSP-16.48 doing in response to ethanol or nicotine? One possibility is that the endogenous protein is acting directly within specific cellular pathways that are most affected by addictive substances through essential protein–protein interactions requiring the seven amino acids (38–44) of the protein’s N terminus. Potential binding partners could be those proteins previously identified in genetic experiments of addiction using model organisms such as mice, *Drosophila*, and *C. elegans* (Davies *et al.* 2003; Feng *et al.* 2006; Offenhauser *et al.* 2006; Rothenfluh *et al.* 2006; Kapfhamer *et al.* 2008; Graham *et al.* 2009; Speca *et al.* 2010; Johnson *et al.* 2013). Small HSPs are known to interact directly with the cytoskeleton (Wettstein *et al.* 2012). Although qualitatively the cytoskeleton of intact animals appeared unaffected by ethanol or nicotine application under the conditions of our experiments, longer-term exposure (hours) of cells in culture can affect the cytoskeleton (Loureiro *et al.* 2011; Gu *et al.* 2013). An alternative hypothesis therefore is that the effects of HSP-16.48 are exacted through cytoskeletal interactions. As there have been no descriptions for specific binding partners for HSP-16.48, these hypotheses would require substantial further investigation.

Overexpression of *hsp-16.48* can dramatically alter ethanol and nicotine sensitivity in the wild type; yet, its overexpression in the *hsf-1sy441* genetic background is statistically beneficial but more limited. This is perhaps not surprising as small HSPs are well known to act in cooperation with other ATP-dependent chaperones, such as the HSP70s, that are downstream of *hsf-1* (Garrido *et al.* 2012). Our RNAi screen indicated that RNAi of *hsp-70* could phenocopy *hsf-1* in ethanol sensitivity, but not nicotine sensitivity. In contrast, RNAi of an alternative HSP70, *F11F1.1*, phenocopied *hsf-1* in nicotine sensitivity only. It is possible that HSP-16.48 is a common factor in drug sensitivity, but associates with divergent HSP70 isoforms dependent upon environmental circumstances. Nevertheless the RNAi and overexpression data demonstrate that *hsp-16.48* is both necessary and sufficient for drug sensitivity phenotypes and indicate that, in a wild-type background at least, *hsp-16.48* expression level is the limiting factor.

The preconditioning HS protocol was found to enhance the effect of the *hsf-1sy441* phenotype in drug sensitivity, which was surprisingly the opposite to the effect of HS in wild-type worms. This effect may be a consequence of the HS protocol itself damaging the temperature-sensitive *hsf-1sy441* strain, as HS did negatively affect endogenous rates of locomotion. Alternatively, this finding may imply an HSF-1-independent effect of HS acting detrimentally in the organism that becomes apparent only in the *loss-of-function hsf-1sy441* strain. If the second hypothesis was true, then the HSF-1-dependent effects of HS in the wild-type worm would be greater than that estimated here.

A recent report indicated that *hsf-1* overexpression could increase thermotolerance and lifespan of *C. elegans* through an upregulation in *pat-10* and a subsequent protection of the cytoskeleton (Baird *et al.* 2014). Our *hsf-1*-dependent drug sensitivity phenotypes appear to be of a different origin for the following reasons. First, overexpression of *hsf-1* does not alter basal sensitivity to either ethanol or nicotine. Second, neither drug upregulates the expression of *pat-10*, whereas temperature stress does. Third, overexpression of *pat-10* does not alter ethanol sensitivity, whereas it does protect against temperature stress. Finally, *pat-10* acts to protect the cytoskeleton in the face of temperature stress and our evidence, at least qualitatively, does not point to an acute effect of ethanol or nicotine on the cytoskeleton.

There is considerable evidence for the conservation of the genetic basis of addiction, from invertebrate models to mammals, including humans. For example the large conductance calcium-activated potassium channel, the voltage-insensitive cation leak channel, and the exocytotic proteins Rab-3 and Munc18 have effects on both ethanol sensitivity in nematodes and have altered complex alcohol phenotypes in mice (Davies *et al.* 2003; Fehr *et al.* 2005; Kapfhamer *et al.* 2008; Graham *et al.* 2009; Speca *et al.* 2010; Johnson *et al.* 2013). Indeed mutations in some of the targets affecting either alcohol or nicotine sensitivity in nematodes have also been identified in GWAS studies, albeit without achieving statistical significance (Bierut *et al.* 2007; Edenberg *et al.* 2010). Although small heat shock proteins have not been previously associated with addiction in GWAS studies, the expression levels of α-crystallin itself is increased in both mice strains with a preference for high alcohol intake (Mulligan *et al.* 2006) and in actual human addicts (Iwamoto *et al.* 2004). Therefore it is highly likely that the results from the nematode presented here have real translational implications for the genetic basis of human addiction.

Genetic risk factors for diverse complex disorders are increasingly found to be linked, such as addiction with cancer (Bierut 2011; Wang *et al.* 2012). Intriguingly the genetics of certain cancers have recently been ascribed to an HSF-1-dependent transcriptional program that is independent of stress (Mendillo *et al.* 2012). Our results indicate that HSF-1 transcriptional activity and downstream HSP effectors within the nervous system specifically can also regulate addiction independent of stress. HSF-1 knockouts in mice show abnormal affective behavior, including increased susceptibility to depression-like behavior and aggression (Uchida *et al.* 2011), and molecular chaperones are associated with a number of human diseases, including neurodegenerative disorders. It is therefore evident that the cellular roles of HSF-1 and HSPs are more intrinsic than as simple stress response proteins. Our research expands upon these functions, potentially implicating HSF-1 and small HSPs as a central intracellular hub linking genetic predisposition to multiple, complex neurological disorders, including susceptibility to addiction. These findings could then facilitate new avenues for pharmacological intervention to addiction in general.
